# Physical capability predicts mortality in late mid-life as well as in old age: Findings from a large British cohort study

**DOI:** 10.1016/j.archger.2017.10.001

**Published:** 2018-01

**Authors:** Victoria L. Keevil, Robert Luben, Shabina Hayat, Avan A. Sayer, Nicholas J. Wareham, Kay-Tee Khaw

**Affiliations:** a Department of Public Health and Primary Care, University of Cambridge, Strangeways Research Laboratory, Wort’s Causeway, Cambridge, CB1 8RN, UK; b Medicine for Older People, Addenbrooke’s Hospital, Cambridge University Hospitals NHS Foundation Trust, Cambridge, CB2 0QQ, UK; c NIHR Newcastle Biomedical Research Centre, Newcastle University and Newcastle upon Tyne Hospitals NHS Foundation Trust, UK; d AGE Research Group, Institute of Neuroscience and Institute for Ageing, Newcastle University, UK; e MRC Epidemiology Unit, University of Cambridge, Institute of Metabolic Science, Addenbrooke’s Biomedical Campus, Cambridge, CB2 0QQ, UK

**Keywords:** Physical performance, Mortality, Epidemiology, Mid-late life

## Abstract

**Introduction:**

Low physical capability predicts mortality, perhaps by association with co-morbidity. However, few studies include participants <70 years old with lower co-morbidity burdens compared to older adults. We examined relationships between usual walking speed (UWS), timed chair stands speed, grip strength, standing balance and all-cause mortality in 8477 participants aged 48–92 years enrolled in the European Prospective Investigation of Cancer-Norfolk study.

**Methods:**

Participants (55.1% female) were followed up for 6.0 years (inter-quartile range 4.6, 7.5). Associations were examined using Cox proportional hazards regression by age-group (<70 years versus ≥70 years) and then in the whole cohort adjusted for age, sex, anthropometry, history of diabetes/stroke/myocardial infarction/cancer, smoking, alcohol intake, socioeconomic status, television viewing time and physical activity.

**Results:**

Age and sex adjusted associations were similar in younger and older participants (P_interaction_ all >0.05) and those with lower physical capability had higher mortality risk. For example, in those <70 years old hazard ratios (95% confidence interval) for mortality in the third, second and lowest sex-specific quartiles of UWS compared to the highest were 1.21 (0.75, 1.96), 2.11 (1.35, 3.28) and 2.91 (1.84, 4.62) and in participants ≥70 years old were 1.19 (0.73, 1.95), 2.09 (1.35, 3.24) and 2.64 (1.73, 4.02) respectively. In the whole cohort, strong associations between all physical capability tests and mortality persisted after multivariable adjustment and after excluding participants with co-morbidity.

**Conclusions:**

Physical capability was independently predictive of future mortality risk with similar associations in late mid-life, when co-morbidity burden is lower, as at older age.

## Highlights

• Physical capability predicts mortality in community-based British men and women.
• Mortality associations are similar in mid- (48–69 years) and late-life (70+ years).
• Co-morbidity burden and shared risk factors did not explain associations.
• Biological mechanisms linking physical capability and mortality could be explored.

## Graphical abstract

**Figure:**
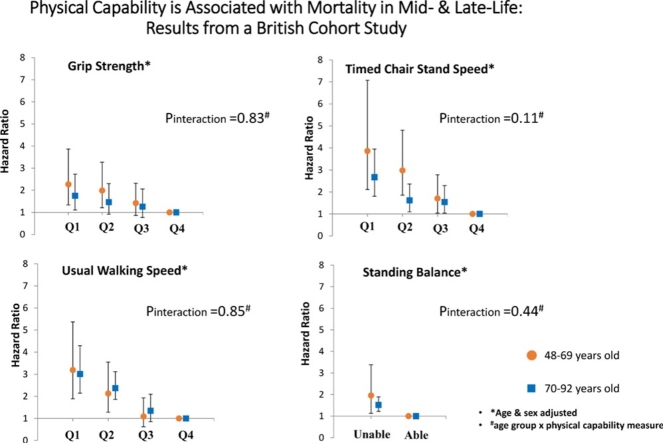

## 1 Introduction

Physical capability, the ability to carry out everyday activities, can be objectively measured using simple tests such as grip strength (GS), timed chair stands speed (TCSS), usual walking speed (UWS) and standing balance (SB). Low performance on these tests has been associated with higher future mortality in both community-based cohorts and patient populations (Wang et al., 2005). In particular, the association between low physical capability and higher mortality has been well described in adults over 70 years old (Cooper, Kuh, & Hardy, 2010; Studenski et al., 2011) with results of a meta-analysis suggesting a linear dose-response relationship (Cooper et al., 2010). This has led to measures such as UWS being termed the sixth ‘vital sign’ of health (Fritz & Lusardi, 2009) and there is growing interest in their use as markers of clinical geriatric syndromes, such as sarcopenia and frailty (Keevil & Romero-Ortuno, 2015).

However, the association between low physical capability and mortality has been less well characterised in adults <70 years old and only a limited number of new reports have been published (Cooper, Strand, Hardy, Patel, & Kuh, 2014; Elbaz et al., 2012, Leong et al., 2015, Ortega and Silventoinen, 2012, Rantanen et al., 2012) since a meta-analysis identified this evidence gap (Cooper et al., 2010). These studies, similar to those included in the previous meta-analysis (Cooper et al., 2010), under-represent women (Elbaz et al., 2012, Ortega and Silventoinen, 2012, Rantanen et al., 2012) and often only evaluate associations between mortality and grip strength rather than exploring a range of physical capability measures (Leong et al., 2015, Ortega and Silventoinen, 2012, Rantanen et al., 2012). Emerging evidence from these studies suggests that the association is weaker in younger adults (Cooper et al., 2010) and that there may be a threshold effect, with only the very lowest performers experiencing increased risk of mortality (Cooper et al., 2014, Elbaz et al., 2012, Katzmarzyk and Craig, 2002, Ortega and Silventoinen, 2012), rather than the linear dose-response relationship described in older adults. A recent meta-analysis of the short physical performance battery (SPPB), which combines performance on UWS, TCS and SB tests, aimed to address part of this evidence gap and did demonstrate a linear relationship between the SPPB score and mortality in a range of community and patient populations in different geographical areas (Pavasini et al., 2016). However, none of the studies included were from the United Kingdom and only 2 included adults with a mean age of <70 years.

If measures of physical capability are to be used in clinical practice it is important to know whether they predict mortality similarly in populations of different ages. Additionally, younger population groups are likely to have lower levels of co-morbidity than older cohorts, an important potential confounding factor in physical capability-mortality associations. Therefore, establishing whether associations differ depending on the age of participants could help us understand why low physical capability predicts mortality. Does physical capability simply reflect the underlying cumulative disease burden of older adults or is there another explanation for its association with mortality?

We used the infrastructure of the European Prospective Investigation of Cancer (EPIC)-Norfolk study to evaluate associations between a range of physical capability measures and mortality in men and women spanning a wide age range (48–92 years old). We hypothesised that if underlying co-morbidity explained the relationship, the association between low physical capability and higher mortality would be weaker in younger compared to older cohort members and a threshold effect may be evident in younger participants.

## 2 Materials and methods

### 2.1 Study population and data collection

At baseline (1993–1997) the EPIC-Norfolk study enrolled over 25 000 community-dwelling men and women (40–70 years old) who were registered with participating GP surgeries in and around the city of Norwich (Norfolk, United Kingdom). This study utilises data from 8477 men and women, now aged 48–92 years old, who underwent tests of physical capability (GS, TCSS, UWS and SB) at the study’s third health examination (3HC, 2006–2011) and had complete follow-up in terms of vital status until January 31st 2015. Full details of the study design have been reported elsewhere (Hayat et al., 2014) and ethical approval was received from the Norfolk Local Research Ethics Committee and the East Norfolk and Waveney NHS Research Governance Committee.

The 3HC was held at a central research clinic. Maximum grip strength was ascertained using a hand-held Smedley Dynamometer (Scandidact, Kvistgaard, Denmark). Participants performed the test standing with their forearms bent at 90 ° and the strongest force (kilograms, kg) generated after two trials in each hand was used. UWS was measured as participants walked a 4 m course at a comfortable pace, using aids if necessary. UWS was calculated by dividing the distance walked by the average time taken out of two attempts (cm/s). TCSS was measured by asking participants to rise from a chair five times as quickly as possible with their arms folded across their chest and their feet flat on the floor. TCSS was calculated by dividing five by the time taken (stands/minute: 60*[5/time, s]). Standing balance was ascertained by asking participants to stand for 10 s with their feet apart in parallel, semi-tandem and then tandem positions. Reasons for non-participation were recorded, identifying those unable to attempt the tests for health reasons.

During the clinic appointment, weight and height were measured using digital scales (to the nearest 0.1 kg, Tanita) and a stadiometer (to the nearest 0.1 cm, Chasmores, UK). Waist circumference (WC) was also measured using a D-loop non-stretch fibreglass tape (to the nearest 0.1 cm) placed around the narrowest point between the ribs and iliac crest (or the level of the umbilicus). The average of two measurements was used.

Additionally, each participant self-reported their smoking status (current, ex-smoker, never smoker), alcohol intake (units/week), current wealth (more than enough, just enough or not enough money), television (TV) viewing (hours/day) and physical activity (active, moderately active, moderately inactive, inactive) by returning a health and lifestyle questionnaire mailed to them with their 3HC clinic appointment. In particular, physical activity was measured using a four point index derived from activity at work, at home and during leisure time, validated against daily energy expenditure (Wareham et al., 2003). Occupational social class had been ascertained at baseline using a similar questionnaire.

A history of heart attack, stroke, cancer (all cancers except non-melanoma skin cancers) and/or diabetes was established by combining self-report of these conditions at baseline (and during the 2HC, 1998- 2000) with incident data captured over the follow-up period via record linkage with hospital episode statistics (International Classification of Disease [ICD] codes: non-fatal MI- ICD9 code 410 and ICD10 codes I21-I22; non-fatal stroke- ICD9 codes 430–438 and ICD10 codes I60-I69; non-fatal cancers- ICD9 codes 140–208 and ICD10 codes C00-C97; diabetes- ICD9 code 250 and ICD10 codes E10-E14). Each co-morbid condition was entered as a separate binary variable in analyses (yes/no).

Participants were followed up from the date of their 3HC clinic appointment until the date of their death or 31st January 2015. The entire cohort has been linked to the NHS Central Register for death and the Office of National Statistics (UK) for death certification since the study’s inception ensuring that no participants were lost to follow-up.

### 2.2 Statistical analyses

Participant characteristics were described using means, medians and proportions by vital status. Relationships between physical capability and all-cause mortality were explored using Kaplan-Meier curves and Cox proportional hazard regression. For these analyses, sex-specific quartiles (Q) of maximum GS and UWS were generated, with the small number of participants who had been unable to undertake the tests for health reasons added to the lowest performance quartile (GS n = 95; UWS n = 45). TCSS ‘quartiles’ were also generated. However, those unable to do the TCS test for health reasons (n = 939) were categorised as the lowest performance ‘quartile’ (Q1) and sex-specific tertiles of TCSS became the upper three ‘quartiles’. The range of each sex-specific category of physical capability are described in Table S1 (Supplementary data). SB was dichotomised into those able versus unable to hold a tandem stand for 10s. Although the standing balance test is usually scored from 0 to 4 depending on ability to stand with feet in a side-by-side and semi-tandem, as well as tandem position (Guralnik et al., 1994), very few members of our cohort were unable to complete the side-by-side and semi-tandem stands. For all physical capability measures the best performance category was chosen as the reference category, so that hazard ratios (HR) represented the risk of mortality associated with lower physical capability.

No interactions between sex and physical capability were identified (GS: p = 0.71; UWS: p = 0.47; TCSS: p = 0.53; SB: p = 0.10) so both sexes were combined in analyses. To check for violations of the proportional hazards (PH) assumption, Kaplan-Meier plots were inspected for each physical capability measure. Additionally, plots of Schoenfeld’s residuals against time were inspected (Schoenfeld, 1982). No violations were identified.

To investigate the possibility of different associations with mortality in younger versus older participants, age and sex adjusted hazard ratios for each physical capability measure (Model 1) were calculated for the whole cohort and after stratification into age-groups (<70 years old versus ≥70 years old). Models with and without an interaction term between age-group and physical capability were compared using likelihood ratio tests.

The potential for co-morbidity or other confounders to explain the relationship between physical capability and mortality was then explored using multivariable models in the whole cohort. First analyses were adjusted for height and weight, in addition to age and sex (Model 2). Then further adjustment for common health risk factors including smoking, alcohol intake, sedentariness (television viewing time), physical activity, occupational social class, and current wealth was made (Model 3) before including the measured co-morbid conditions (Model 4).

Fifteen percent of participants had missing data in at least one co-variable: height (n = 17), waist circumference (n = 19), weight (n = 11), physical activity (n = 120), wealth (n = 483), social class (n = 79), alcohol intake (n = 321) and television viewing (n = 737). Missing data were more likely in those who were female, older, in the lowest physical capability quartile at the 3HC and those who died during the follow-up period (Supplementary data, Table S2). Multiple imputation commands in Stata (version 12.0) were used to impute co-variable data. Imputation models contained all the physical capability measures, all co-variables included in Model 4 and two parameters describing the outcome, namely the variable describing the event (dead) and an estimate of the cumulative hazard from baseline (the Nelson-Aelen estimator). *A priori* imputation models were also stratified by age-group (<70 years old; ≥70 years old) and sex. Twenty imputed datasets were created and Cox regression analyses were run across each imputed dataset with estimates of the HRs combined using Rubin’s rules.

In supplementary work, age and sex adjusted associations were also explored after stratification of the cohort by levels of key potential confounders. For these analyses, grip strength, UWS and TCSS were divided into two categories of good versus poor performance, based on their median cohort values (weak grip strength: <25 kg [women], <40 kg [men]; slow UWS <110 cm/s; slow TCSS <25stands/min). Multivariable analyses were also repeated after excluding those who died in the first year of follow-up and after restricting the sample to those with complete co-variable data only.

## 3 Results

### 3.1 Main findings

The characteristics of the 8477 participants (55.1% female; mean age 68.7 years [sd 8.1]) included in this study are detailed in Table 1. Participants were followed up for a median time of 6.0 years (IQR 4.6, 7.5) during which 642 participants died (162 deaths in those <70 years old and 480 deaths in those ≥70 years old). The crude death rate in men and women respectively was 16.9/1000 person-years (95% CI 15.3, 18.7) and 9.2/1000 person-years (95% CI 8.1, 10.3). Those who died during the follow up period were less likely to consume moderate amounts of alcohol and more likely to be men, older, smokers, have high waist circumference, more co-morbidity, lower physical activity, lower physical capability and to have spent more time watching television at the 3HC than those still alive at the end of the study (Table 1).

**Table 1:** Characteristics of the 8477 participants with physical capability measurements and complete follow-up.

Co-variable mean (SD)^a^^,^^b^	Dead (n = 642)	Alive (n = 7835)	P value
Sex, % male (n)	59.3 (381)	43.7 (3423)	<0.001
Age, years	75.5 (7.8)	68.1 (7.8)	<0.001
Height, cm	166.3 (9.5)	166.3 (9.1)	0.94
Weight, kg	75.3 (15.2)	74.4 (14.1)	0.10
Waist Circumference, cm	98.2 (12.7)	94.2 (12.2)	<0.001
Grip Strength, kg	29.3 (9.7)	31.1 (10.1)	<0.001
Usual Walking Speed, cm/s	93.3 (24.7)	111.3 (24.7)	<0.001
Timed Chair Stands Speed, stands/min	23.3 (7.25)	26.6 (8.2)	<0.001
Standing Balance, % able (n)	71.3 (458)	88.5 (6933)	<0.001

Co-morbidity, % (n)			
Yes	42.5 (273)	15.5 (1215)	
No	57.5 (369)	84.5 (6620)	<0.001

Social Class, % (n)			
Manual	31.5 (202)	33.9 (2655)	
Non-manual	67.5 (433)	65.2 (5108)	0.22

Wealth, % (n)			
Not enough money	5.0 (32)	6.0 (467)	
Just enough money	63.9 (410)	62.6 (4908)	
More than enough money	23.1 (148)	25.9 (2029)	0.27

Physical Activity, % (n)			
Inactive	54.7 (351)	35.1 (2749)	
Moderately inactive	20.1 (129)	29.4 (2306)	
Moderately active	14.2 (91)	17.9 (1400)	
Active	8.3 (53)	16.3 (1278)	<0.001

Daily TV Viewing Time, % (n)			
≥4 h/day	40.5 (260)	31.7 (2481)	
3 < 4 h/day	20.2 (130)	22.4 (1757)	
2 < 3 h/day	13.9 (89)	20.9 (1640)	
<2 h/day	11.8 (76)	16.7 (1307)	<0.001

Smoking habit, % (n)			
Current	5.5 (35)	4.2 (330)	
Ex	55.1 (354)	44.5 (3487)	
Never	36.6 (235)	50.0 (3916)	<0.001

Alcohol Intake, median (IQR)			
None	32.9 (211)	28.4 (2224)	
1–14 units/week	50.6 (325)	57.1 (4476)	
>14 units/week	11.2 (72)	10.8 (848)	0.01

SD: standard deviation; IQR: inter-quartile range.

a Unless otherwise indicated.

b Where applicable percentages may not add up to 100% due to missing data.

**Fig. 1:**
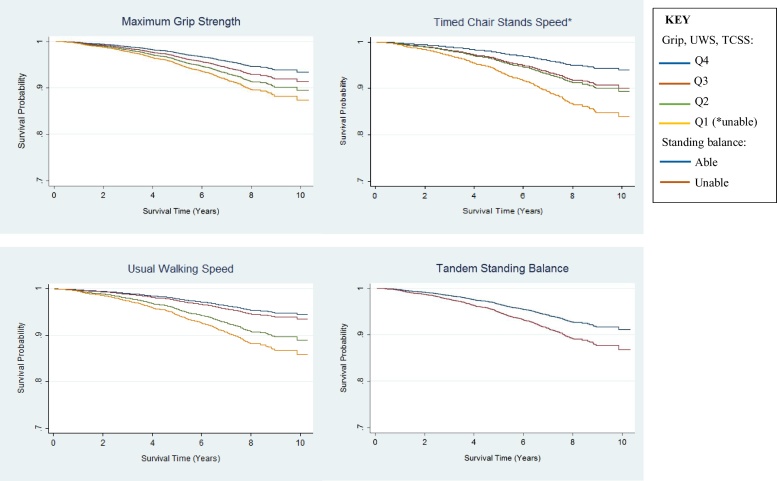
Cox Survival Curves (age and sex adjusted).

Participants in lower sex-specific physical capability categories were more likely to die in the follow up period than those in the highest categories, irrespective of the physical capability measure used (Fig. 1; also see Supplementary data, Table S3 for crude death rates by physical capability level).

Associations persisted after stratification of the cohort into younger (<70 years old) and older (≥70 years old) age groups (Table 2). There was no evidence of effect modification by age and associations between physical capability and mortality were similar in the two age-groups studied (Table 2).

**Table 2:** Associations between objectively measured physical capability and mortality in the EPIC-Norfolk cohort over 6 years of follow-up. Associations are presented for the whole cohort and after division of the cohort into younger (<70 years) and older (≥70 years) age-groups.

			Hazard ratio (95% Confidence Interval)^b^							
			Whole Cohort			<70years old			≥70 years old	
Physical Capability	N	Deaths	Model 1	N	Deaths	Model 1	N	Deaths	Model 1	P_interaction_^c^
Grip										
Q1	2136	290	1.97 (1.47, 2.64)	693	30	1.87 (1.15, 3.02)	1443	260	1.94 (1.28, 2.95)	
Q2	2038	173	1.63 (1.22, 2.19)	1017	45	1.89 (1.23, 2.91)	1021	128	1.57 (1.02, 2.40)	
Q3	2085	112	1.33 (0.98, 1.80)	1384	46	1.37 (0.90, 2.09)	701	66	1.32 (0.84, 2.08)	
Q4	2218	67	1.00	1801	41	1.00	417	26	1.00	0.82

TCSS										
Q1^a^	939	173	2.80 (2.11, 3.71)	307	20	3.02 (1.77, 5.14)	632	153	2.65 (1.84, 3.81)	
Q2	2512	219	1.78 (1.37, 2.32)	1093	48	2.21 (1.45, 3.34)	1419	171	1.66 (1.17, 2.36)	
Q3	2504	167	1.66 (1.27, 2.17)	1527	50	1.54 (1.02, 2.31)	977	117	1.72 (1.20, 2.48)	
Q4	2522	83	1.00	1968	44	1.00	554	39	1.00	0.99

UWS										
Q1	2152	326	2.67 (1.98, 3.61)	691	44	2.91 (1.84, 4.62)	1461	282	2.64 (1.73, 4.02)	
Q2	2096	177	2.07 (1.52, 2.81)	1136	52	2.11 (1.35, 3.28)	960	125	2.09 (1.35, 3.24)	
Q3	2103	83	1.19 (0.84, 1.67)	1396	34	1.21 (0.75, 1.96)	707	49	1.19 (0.73, 1.95)	
Q4	2126	56	1.00	1672	32	1.00	454	24	1.00	0.40

Standing Balance										
Unable	1086	184	1.52 (1.26, 1.82)	288	18	1.83 (1.11, 2.99)	798	166	1.45 (1.18, 1.77)	
Able	7391	458	1.00	4607	144	1.00	2784	314	1.00	0.47

a Those unable to do the test for health reasons.

b Adjusted for age and sex.

c P value for age-group#physical capability interaction term.

Adjustment for a range of health risk factors did not fully explain the observed associations (Table 3) and associations also persisted after further adjustment for the co-morbid conditions considered. Additionally, associations persisted within strata of important confounders, including sex, waist circumference, physical activity, TV viewing time, smoking, alcohol intake and co-morbidity (Supplementary data, Table S4). For example, in those without any history of cancer, stroke, diabetes or myocardial infarction at the 3HC (n = 6989) the risk of mortality was 1.32 (95% CI 1.03, 1.68), 1.77 (95% CI 1.38, 2.27), 1.90 (95% CI 1.48, 2.43) and 1.39 (95% CI 1.08, 1.79) times greater in those with weak GS, slow TCSS, slow UWS and poor SB compared to good performers.

**Table 3:** Associations between physical capability and mortality after multivariable adjustment.

			Hazard ratio (95% Confidence Interval)^b^		
Physical Capability	N	Deaths	Model 2 (age, sex, body size)	Model 3 (all except co-morbidity)	Model 4 (+co-morbidity)
Grip					
Q1	2136	290	2.00 (1.49, 2.70)	1.84 (1.36, 2.49)	1.80 (1.33, 2.43)
Q2	2038	173	1.65 (1.23, 2.22)	1.61 (1.20, 2.17)	1.50 (1.12, 2.03)
Q3	2085	112	1.35 (0.99, 1.83)	1.30 (0.96, 1.77)	1.29 (0.95, 1.75)
Q4	2218	67	1.00	1.00	1.00

TCSS					
Q1^a^	939	173	2.84 (2.12, 3.78)	2.46 (1.83, 3.31)	2.29 (1.70, 3.09)
Q2	2512	219	1.81 (1.39, 2.37)	1.65 (1.26, 2.16)	1.55 (1.18, 2.03)
Q3	2504	167	1.68 (1.29, 2.19)	1.59 (1.22, 2.08)	1.56 (1.19, 2.04)
Q4	2522	83	1.00	1.00	1.00

UWS					
Q1	2152	326	2.63 (1.93, 3.56)	2.35 (1.72, 3.19)	2.16 (1.59, 2.95)
Q2	2096	177	2.05 (1.51, 2.78)	1.94 (1.43, 2.64)	1.86 (1.36, 2.53)
Q3	2103	83	1.18 (0.84, 1.66)	1.17 (0.83, 1.65)	1.16 (0.83, 1.63)
Q4	2126	56	1.00	1.00	1.00

SB					
Unable	1086	184	1.49 (1.24, 1.79)	1.37 (1.14, 1.66)	1.33 (1.10, 1.61)
Able	7391	458	1.00	1.00	1.00

a Those unable to do the test for health reasons.

b Model 2: adjusted for age, sex, height & weight. Model 3: adjusted for model 2 + social class, wealth, smoking status, alcohol intake, waist circumference (WC), television (TV) viewing time, physical activity, Model 4: adjusted for model 3 + history of cancer (excluding non-melanoma skin cancer), diabetes, stroke and heart attack.

### 3.2 Sensitivity analyses

Analyses were conducted after exclusion of participants who died in the first year of follow up (n = 8433, deaths = 598) and after exclusion of participants with incomplete co-variable data (n = 7168, deaths = 504). These did not alter the results (results available on request).

## 4 Discussion

Lower physical capability was consistently associated with higher mortality in >8000 community-based British men and women and a dose-response relationship was evident regardless of age.

Lower physical capability has been robustly associated with higher mortality in people >70 years old (Cooper et al., 2010, Studenski et al., 2011) but few studies have included younger adults and those that have suggest the association might differ. Only those in the lowest quintile of performance (or unable to participate in the tests) were at a higher risk of death in a British cohort of men and women aged 53 years suggesting a threshold effect (Cooper et al., 2014). Additionally, non-linear associations were observed between lower grip strength and higher mortality in Swedish adolescent males (Ortega & Silventoinen, 2012), with those in the lowest tenth of muscle strength at particularly high risk, and no strong associations between grip strength and mortality were observed in 20–69 year old Canadian men and women (Katzmarzyk & Craig, 2002).

The underlying mechanisms associating lower physical capability with higher mortality are debated. One theory suggests that residual confounding from unmeasured or sub-clinical disease could explain the association. If this were the case, it might be expected that associations would be weaker in younger adults who have a lower co-morbidity burden or only evident in those younger adults most physically unable, who are more likely to have significant illness. However, we observed no evidence of differing associations between physical capability and mortality by age-group and neither adjustment for co-morbidity, exclusion of participants with at least one baseline co-morbid disease nor exclusion of those who died in the first year of follow-up attenuated relationships. Additionally, our results agree with the few other reports that have examined whether age modifies the association between physical capability and mortality. For example, the association between UWS and mortality in British civil servants aged 50–73 years was not modified by age (Elbaz et al., 2012) and linear associations between grip strength and mortality were observed across all age-groups within a Japanese cohort aged 35–74 years (Sasaki, Kasagi, Yamada, & Fujita, 2007).

We also observed that associations were not substantially attenuated after adjustment for several common health determinants, reducing the likelihood that associations could be explained by risk factors shared between low physical capability and high mortality. In other words, although central obesity, sedentary behaviour, low physical activity and several other health and lifestyle factors included in analyses are associated both with low physical capability (Cooper, Mishra, & Kuh, 2011; Keevil et al., 2014) and higher mortality (Khaw et al., 2006, Wijndaele et al., 2011), they could not fully account for the associations between physical capability and mortality.

If underlying co-morbidity or exposure to shared risk factors cannot fully explain the relationship between physical capability and mortality, it is possible that other biological mechanisms explain the association. Investigators from the Baltimore Longitudinal Study of Ageing suggest that an imbalance of energy availability and energy requirement in later life, leading to an energy deficit, could explain the ‘slowing up’ associated with age-related organism fragility (Schrack, Simonsick, & Ferrucci, 2011). They hypothesise that when this energy imbalance reaches a critical level, such that basic metabolic functions are threatened, the organism compensates by diverting energy spent on other tasks (e.g., mobility) to support essential metabolic processes. Consistent with this theory, the energy required to walk at the same sub-maximal pace (0.67 m/s) increases with age whilst the total available energy (maximal energy expenditure, VO2_max_ − resting metabolic rate, RMR) declines (Schrack et al., 2011), providing evidence for a less efficient metabolic system in older age. Furthermore, older adults whose RMR does not decline in parallel with the age-related loss of lean mass have higher mortality (Ruggiero et al., 2008).

There are several limitations to this study. Some important co-morbidities e.g., arthritis, were not included because relevant data had not been collected. Furthermore, it is possible that analyses were underpowered to detect interactions between age and physical capability since there were fewer deaths in younger participants. This also limited the number of sub-groups based on age that could be considered. Therefore, our conclusions must be cautious and the work presented here should be repeated when more follow-up time has elapsed. Additionally, our measure of standing balance was not able to discern significant heterogeneity amongst the cohort because of a ‘floor’ effect. Most cohort members could fully complete the easier parts of the test and only tandem stands provided evidence of differing balance function.

However, most studies to date have limited power and it is possible that the ‘threshold effect’ observed in some studies of younger adults is a consequence of relatively few deaths in those above the performance ‘threshold’ identified. Additionally our work has several strengths. We evaluated several different physical capability tests measured in a large cohort of community-based men and women spanning a wide age-range, including participants younger than 70 years old. We excluded very few participants from analyses since vital status was ascertained for all cohort members, reasons for non-participation in the physical capability tests were recorded and we used multiple imputation to account for missing co-variable data.

## 5 Conclusion

In summary, physical capability predicted mortality in late mid-life, when the co-morbidity burden is lower, as well as in old age. Future studies should explore underlying biological pathways linking low physical capability to increased organism fragility.

## Conflicts of Interest

None.

## Funding

This work was supported by a Wellcome Trust clinical training fellowship awarded to Victoria Keevil [092077/Z/10/Z] and by programme grants from the Medical Research Council (MRC) (grant numbers G9502233, G0401527), Cancer Research UK (grant number C864/A8257) and Research into Ageing (grant number 262). NJW is also supported by the MRC (grant numbers MC_UU_12015/3, MC_UU_12015/4).
