# Supplementary material for: Physical capability predicts mortality in late mid-life as well as in old age: Findings from a large British cohort study
Source: Arch Gerontol Geriatr. 2018 Jan;74:77–82. doi: 10.1016/j.archger.2017.10.001 (PMC5701965; doi:10.1016/j.archger.2017.10.001)
Supplement: Supplementary file 1 [file mmc1.docx]

**Supplementary Data**

Table S1: The range of physical capability in sex-specific categories of grip strength, usual walking speed & timed chair stands speed.

|  | **Range** | |
| --- | --- | --- |
| **Physical Capability** | **Men** | **Women** |
| **Grip Strength (kg)** |  |  |
| Q1 | Unable or 9.0 - 33.0 | Unable or 3.0 - 20.5 |
| Q2 | 33.5 - 38.5 | 21.0 - 24.0 |
| Q3 | 39.0 - 44.5 | 24.5 - 27.9 |
| Q4 | 45.0 - 72.0 | 28.0 - 44.0 |
| **Usual Walking Speed (cm/s)** |  |  |
| Q1 | Unable or 13.7 - 95.9 | Unable or 13.1 - 92.4 |
| Q2 | 96.0 - 111.4 | 92.5 - 108.8 |
| Q3 | 111.5 - 127.4 | 109.0 - 124.6 |
| Q4 | 127.4 - 216.2 | 124.8 - 215.1 |
| **Timed Chair Stands Speed (stands/min)** |  |  |
| Q1 | Unable | Unable |
| Q2 | 5.0 – 22.6 | 5.2 – 22.1 |
| Q3 | 22.7 – 29.1 | 22.2 – 28.3 |
| Q4 | 29.2 – 73.2 | 28.4 – 71.9 |

Table S2: Characteristics of participants with complete and incomplete co-variable data

| **Co-variable** | **Complete Data**  **(n=7168)** | **Missing Data**  **(n=1309)** | **P value** |
| --- | --- | --- | --- |
| **Age**, years (mean, SD) | 68.3 (8.0) | 71.1 (8.0) | <0.001 |
| **Sex, %(n)**  Male | 45.9 (3290) | 39.3 (514) | <0.001 |
| **Vital Status, %(n)**  Dead | 5.7 (407) | 8.2 (107) | 0.001 |
| **Grip Strength, %(n)**  Lowest Quartile | 23.6 (1694) | 33.8 (442) | <0.001 |
| **UWS, %(n)**  Lowest Quartile | 23.7 (1701) | 34.5 (451) | <0.001 |
| **TCSS, %(n)**  Unable | 10.0 (720) | 16.7 (219) | <0.001 |
| **Standing Balance, %(n)**  Unable | 11.8 (848) | 18.2 (238) | <0.001 |

UWS: usual walking speed; TCSS: timed chair stands speed

Table S3: Crude death rates in the EPIC-Norfolk cohort.

|  | **Crude Death Rate per 1000 person-years (95% Confidence Interval)** | | | | | | | | |
| --- | --- | --- | --- | --- | --- | --- | --- | --- | --- |
|  | **Whole Cohort** | | | **<70years old** | | | **>70years old** | | |
| **Physical Capability** | **Person-years** | **Deaths** | **Death Rate** | **Person-years** | **Deaths** | **Death Rate** | **Person-years** | **Deaths** | **Death Rate** |
| **Grip** |  |  |  |  |  |  |  |  |  |
| **Q1** | 12043 | 290 | 24.1 (21.5, 27.0) | 4086 | 30 | 7.3 (5.1, 10.5) | 7957 | 260 | 32.7 (28.9, 36.9) |
| **Q2** | 12041 | 173 | 14.4 (12.4, 16.7) | 6078 | 45 | 7.4 (5.5, 9.9) | 5964 | 128 | 21.5 (18.0, 25.5) |
| **Q3** | 12630 | 112 | 8.6 (7.4, 10.7) | 8505 | 46 | 5.4 (4.0, 7.2) | 4125 | 66 | 16.0 (12.6, 20.4) |
| **Q4** | 14358 | 67 | 4.7 (3.7, 5.9) | 11741 | 41 | 3.5 (2.5, 4.7) | 2617 | 26 | 9.9 (6.7, 14.6) |
| **TCSS** |  |  |  |  |  |  |  |  |  |
| **Q1*** | 5384 | 173 | 32.1 (27.7, 37.3) | 1877 | 20 | 10.7 (6.9, 16.5) | 3507 | 153 | 43.6 (37.2, 51.1) |
| **Q2** | 13985 | 219 | 15.7 (13.7, 17.8) | 6131 | 48 | 7.8 (5.9, 10.4) | 7853 | 117 | 21.8 (18.7, 26.0) |
| **Q3** | 15074 | 167 | 11.1 (9.5, 12.9) | 9305 | 50 | 5.4 (4.1, 7.1) | 5769 | 117 | 20.3 (16.9, 24.3) |
| **Q4** | 16629 | 83 | 5.0 (4.0, 6.2) | 13098 | 44 | 3.4 (2.5, 4.5) | 3531 | 39 | 11.0 (8.1, 15.1) |
| **UWS** |  |  |  |  |  |  |  |  |  |
| **Q1** | 12523 | 326 | 26.0 (23.4, 29.0) | 4221 | 44 | 10.4 (7.8, 14.1) | 8302 | 282 | 34.0 (30.2, 38.1) |
| **Q2** | 12845 | 177 | 14.0 (12.1, 16.2) | 7090 | 52 | 7.3 (5.6, 9.6) | 5573 | 125 | 22.4 (18.8, 26.7) |
| **Q3** | 12663 | 83 | 6.5 (5.2, 8.0) | 8698 | 34 | 3.9 (2.8, 5.5) | 4147 | 49 | 11.8 (8.9, 26.7) |
| **Q4** | 12523 | 56 | 4.3 (3.3, 5.6) | 10402 | 32 | 3.1 (2.2, 4.4) | 2639 | 24 | 9.1 (6.1, 13.6) |
| **Standing Balance** |  |  |  |  |  |  |  |  |  |
| **Unable** | 6317 | 184 | 29.1 (25.2, 33.7) | 1822 | 18 | 9.9 (6.2, 15.7) | 4496 | 166 | 36.9 (31.7, 43.0) |
| **Able** | 44756 | 458 | 10.2 (9.3, 11.2) | 28590 | 144 | 5.0 (4.3, 5.9) | 16166 | 314 | 19.4 (17.4, 21.7) |

*those unable to do the test for health reasons

Table S4: Associations between mortality and physical capability after stratification by level of confounding variables.

|  |  |  | **Hazard Ratio (95% Confidence Interval)*** | | | | | | | | | | |
| --- | --- | --- | --- | --- | --- | --- | --- | --- | --- | --- | --- | --- | --- |
|  |  |  | **Maximum Grip Strength**** | | | **Timed Chair Stands Speed**** | | | **Usual Walking Speed**** | | | **Standing Balance**** | |
| **Co-variable** | **N^#^** | **Deaths^#^** | **Strong** | **Weak** | **Fast** | | **Slow** | **Fast** | | **Slow** | **Able** | | **Unable** |
| **Age** |  |  |  |  |  | |  |  | |  |  | |  |
| <70 years | 4895 | 162 | 1.00 | 1.55 (1.13, 2.12) | 1.00 | | 1.94 (1.41, 2.66) | 1.00 | | 2.14 (1.56, 2.93) | 1.00 | | 1.83 (1.11, 2.99) |
| >70 years | 3582 | 480 | 1.00 | 1.50 (1.17, 1.93) | 1.00 | | 1.63 (1.29, 2.07) | 1.00 | | 2.02 (1.58, 2.58) | 1.00 | | 1.45 (1.18, 1.77) |
| **Sex** |  |  |  |  |  | |  |  | |  |  | |  |
| Men | 3804 | 381 | 1.00 | 1.33 (1.03, 1.71) | 1.00 | | 1.89 (1.47, 2.42) | 1.00 | | 1.91 (1.50, 2.44) | 1.00 | | 1.70 (1.33, 2.17) |
| Women | 4673 | 261 | 1.00 | 1.79 (1.32, 2.43) | 1.00 | | 1.51 (1.13, 2.03) | 1.00 | | 2.26 (1.64, 3.12) | 1.00 | | 1.30 (0.98, 1.72) |
| **Co-morbidity** |  |  |  |  |  | |  |  | |  |  | |  |
| Any | 1488 | 273 | 1.00 | 1.63 (1.19, 2.25) | 1.00 | | 1.43 (1.07, 1.92) | 1.00 | | 1.86 (1.36, 2.54) | 1.00 | | 1.49 (1.14, 1.96) |
| None | 6989 | 369 | 1.00 | 1.32 (1.03, 1.68) | 1.00 | | 1.77 (1.38, 2.27) | 1.00 | | 1.90 (1.48, 2.43) | 1.00 | | 1.39 (1.08, 1.79) |
| **Smoking** |  |  |  |  |  | |  |  | |  |  | |  |
| Current/ Ex | 4206 | 389 | 1.00 | 1.53 (1.19, 1.96) | 1.00 | | 1.84 (1.44, 2.36) | 1.00 | | 1.75 (1.37, 2.24) | 1.00 | | 1.42 (1.12, 1.79) |
| Never | 4151 | 235 | 1.00 | 1.55 (1.13, 2.13) | 1.00 | | 1.58 (1.16, 2.14) | 1.00 | | 2.50 (1.82, 3.44) | 1.00 | | 1.70 (1.25, 2.31) |
| **Social Class** |  |  |  |  |  | |  |  | |  |  | |  |
| Manual | 2857 | 202 | 1.00 | 1.41 (1.00, 1.99) | 1.00 | | 1.38 (1.00, 1.90) | 1.00 | | 1.79 (1.27, 2.52) | 1.00 | | 1.11 (0.78, 1.58) |
| Non-manual | 5541 | 433 | 1.00 | 1.59 (1.25, 2.02) | 1.00 | | 1.94 (1.53, 2.45) | 1.00 | | 2.19 (1.73, 2.77) | 1.00 | | 1.69 (1.35, 2.10) |
| **Alcohol** |  |  |  |  |  | |  |  | |  |  | |  |
| None | 2435 | 211 | 1.00 | 1.54 (1.09, 2.17) | 1.00 | | 1.74 (1.23, 2.44) | 1.00 | | 1.99 (1.39, 2.85) | 1.00 | | 2.04 (1.51, 2.75) |
| >1 unit/week | 5721 | 397 | 1.00 | 1.47 (1.16, 1.88) | 1.00 | | 1.69 (1.34, 2.14) | 1.00 | | 2.03 (1.60, 2.57) | 1.00 | | 1.26 (0.98, 1.62) |
| **Waist Circumference***** |  |  |  |  |  | |  |  | |  |  | |  |
| High | 3996 | 333 | 1.00 | 1.50 (1.14, 1.96) | 1.00 | | 1.71 (1.28, 2.30) | 1.00 | | 1.87 (1.39, 2.52) | 1.00 | | 1.58 (1.24, 2.02) |
| Low | 4462 | 305 | 1.00 | 1.52 (1.14, 2.02) | 1.00 | | 1.67 (1.30, 2.16) | 1.00 | | 2.10 (1.62, 2.72) | 1.00 | | 1.39 (1.04, 1.85) |
| **TV viewing time** |  |  |  |  |  | |  |  | |  |  | |  |
| >4 hours/day | 2741 | 260 | 1.00 | 1.76 (1.29, 2.41) | 1.00 | | 1.83 (1.34, 2.50) | 1.00 | | 1.67 (1.22, 2.27) | 1.00 | | 1.08 (0.81, 1.46) |
| <4 hours/day | 4999 | 295 | 1.00 | 1.34 (1.02, 1.77) | 1.00 | | 1.51 (1.16, 1.98) | 1.00 | | 2.11 (1.61, 2.78) | 1.00 | | 1.88 (1.44, 2.47) |
| **Physical Activity** |  |  |  |  |  | |  |  | |  |  | |  |
| Inactive | 5535 | 480 | 1.00 | 1.77 (1.40, 2.25) | 1.00 | | 1.76 (1.40, 2.22) | 1.00 | | 2.05 (1.62, 2.59) | 1.00 | | 1.48 (1.20, 1.83) |
| Active | 2822 | 144 | 1.00 | 1.00 (0.69, 1.45) | 1.00 | | 1.63 (1.14, 2.32) | 1.00 | | 1.99 (1.38, 2.88) | 1.00 | | 1.70 (1.11, 2.59) |

**^#^**Note that for some variables the numbers in each strata will not add up to the total number of participants/ events in this study due to missing data. *Adjusted for age and sex at the 3HC. **Weak grip strength was defined as <25kg (women) or <40kg (men) , slow timed chair stands speed was defined as <25 stands/ minute (this equates to completing 5 chair rises in >12 seconds), slow usual walking speed was defined as <110cm/s and poor standing balance as those unable to hold a tandem stand for 10 seconds. ***High waist circumference: >88cm (women) or >102cm (men); low waist circumference <102cm (men) or <88cm (women). Note that the four point physical activity score was collapsed into two levels (inactive/ moderately inactive & active/ moderately active)
